# Differential effects of minocycline on human breast epithelial cells, human breast cancer cells and their tumor hybrids

**DOI:** 10.1007/s11033-025-10666-1

**Published:** 2025-06-05

**Authors:** Fuad Moayed, Silvia Keil, Thomas Dittmar, Julian Weiler

**Affiliations:** Institute of Immunology, Center for Biomedical Education and Research (ZBAF), Witten/Herdecke University, Stockumer Str. 10, Witten, Germany

**Keywords:** Breast cancer, Cell fusion, Minocycline

## Abstract

**Background:**

The antibiotic minocycline has been suggested as a potential agent in cancer therapy due to its anti-inflammatory properties and effectiveness as an NF-κB inhibitor. In previous studies, we showed that minocycline could effectively block the fusion of breast epithelial cells and cancer cells. However, its influence on breast cancer cell characteristics, including proliferation, migration, and gene expression has not yet been investigated.

**Methods:**

M13SV1-EGFP-Neo breast epithelial cells, HS578T-Hyg breast cancer cells and M13HS-2 and M13HS-8 tumor hybrids were used as breast (cancer) model cell lines in this study. Cells were treated with up to 50 µg/ml minocycline. An XTT assay and a colony formation assay were used to study cell proliferation. Western blot analysis and Zymography were used to examine the expression of MMP-2 and MMP-9, EMT, and stemness marker. Cell migration was measured by Scratch assay. Using a two-way ANOVA and the Tukey post-hoc test, statistical significance was determined.

**Results:**

Minocycline inhibited proliferation and colony formation capacity in a dose-dependent manner, whereas EMT and stemness marker expression remained unchanged in all cell lines. Zymography data showed that MMP-2 and MMP-9 expression was down-regulated M13SV1-EGFP-Neo treated with minocycline, but not in HS578T-Hyg cells or M13HS-2 and M13HS-8 tumor hybrids. Minocycline inhibited the migration of M13SV1-EGFP-Neo cells in a dose-dependent manner, while the migration of HS578T-Hyg, M13HS-2 and M13HS-8 tumor hybrid cells necessitated a minimum of 25 µg/ml minocycline,

**Conclusions:**

The results showed that non-malignant cells and neoplastic cells reacted differently to minocycline. This could mean that minocycline will have unwanted side effects if it is used in cancer therapy.

**Supplementary Information:**

The online version contains supplementary material available at 10.1007/s11033-025-10666-1.

## Introduction

Minocycline is a well-known second-generation tetracycline derivative [1, 2]. Apart from its widespread application as an antibiotic, minocycline possesses anti-inflammatory, neuroprotective, and even anti-tumorigenic characteristics. These attributes bolster the possibility that minocycline could find application in the treatment of neurodegenerative diseases and cancer [1–4]. Minocycline has been shown to have anti-cancer molecular mechanisms against a variety of cancer types, including ovarian cancer, breast cancer, glioma, liver cancer, melanoma, and colorectal cancer [2, 5–8]. In addition to the induction of apoptosis [7, 9], minocycline also prevented cancer cells from proliferating by stopping their cell cycle [7, 9, 10]. It also successfully suppressed the nuclear factor-kappa-light-chain-enhancer of activated B cells (NF-κB) pathway, which was accompanied by a decrease in the expression of matrix metalloproteinases (MMP), such as MMP-2 and MMP-9 as well as inflammatory cytokines like tumor necrosis factor-α (TNF-α) and interleukin-1β (IL-1β) [11]. Likewise, Yang et al. found that minocycline directly binds the Src kinase Lyn, thereby inactivating signal transducer and activator of transcription 3 (STAT3) signaling concomitant with suppression of epithelial-to-mesenchymal transition (EMT) and colorectal cancer metastasis [8]. These results suggest that minocycline interferes with the transcription machinery in (cancer) cells. This might also hold true for apoptosis triggered by minocycline. Here, minocycline increased the expression of apoptotic genes like caspase-3, -8 and − 9, while suppressing the expression of the anti-apoptotic genes Bcl2 and Bcl-xl [2, 7].

Although there is a growing body of evidence suggesting that minocycline may be effective in cancer therapy, there have been only a handful of studies published thus far that explore its potential use in breast cancer [5, 10–12]. Niu and colleagues demonstrated that the combined use of celecoxib and minocycline had better inhibitory effects on the osseous metastasis of breast cancer, compared to celecoxib or minocycline alone. This was attributed to increased tumor-cell death and decreased tumor-derived MMP-9 and vascular endothelial growth factor expression [5]. A more recent study using MCF-7 breast cancer cells revealed that minocycline effectively induced apoptosis in MCF-7 cells in a dose-dependent manner and, similar to Niu et al., it also reduced the migratory potential of the cells by blocking MMP-2 and MMP-9 activity [12]. We have demonstrated that minocycline inhibited the expression of fusion-relevant factors and suppressed the transcriptional activity of NF-κB [10, 11]. This resulted in the inhibition of the TNF-α induced fusion of M13SV1-Cre human breast epithelial cells and MDA-MB-435-pFDR1 cancer cells [10, 11].

Cell fusion is a fundamental biological phenomenon and describes the merger of two and more cells, which is inevitable for physiological processes, such as fertilization, placentation, myogenesis, osteoclastogenesis and wound healing [13–16]. Moreover, membrane merger and cell fusion also occur in the infection of host cells with enveloped viruses, as well as in cancer [13–16]. In cancer, it is presumed that the fusion of cancer cells and normal cells, such as macrophages and stem cells could result in tumor hybrids. These hybrids could possess novel properties, such as an enhanced metastatogenic capacity, an increased drug resistance, and even cancer stem cell properties [15–18]. Indeed, tumor hybrids have actually been detected in primary tumors as well as in the circulation of different solid cancers like pancreatic, colorectal, breast and uveal melanoma [19–22]. Moreover, high circulating tumor hybrid numbers were linked to a poor prognosis in pancreatic cancer [20] and metastatic progression in uveal melanoma [21]. According to these findings, tumor hybrids are probably involved in cancer progression, which means that new targeted anti-cancer treatments may be able to target them. We have demonstrated that minocycline not only suppressed the TNF-α-induced fusion of M13SV1-Cre human breast epithelial cells and MDA-MB-435-pFDR1 cancer cells, but also impaired the proliferation of both cell types in a dose-dependent manner [10, 11]. Consequently, we investigated the potential effects of minocycline on M13HS tumor hybrids, which were formed through spontaneous fusion events between M13SV1-EGFP-Neo human breast epithelial cells and HS578T-Hyg human breast cancer cells [23]. Here, we investigated the influence of minocycline on cell proliferation, colony formation, the expression of EMT markers, MMP-2 and MMP-9, and cell migration.

## Materials and methods

### Cell culture

M13SV1-EGFP-Neo, HS578T-Hyg and M13HS-2 and M13HS-8 cells were generated and cultivated as described [23]. In brief, M13SV1-EGFP-Neo human breast epithelial cells were created by stable transfection with the p-enhanced green fluorescent protein (EGFP)-Neo vector from M13SV1 cells (kindly provided by James Trosko, Michigan State University, East Lansing, MI, USA [24]) [23]. HS578T-Hyg human breast cancer cells were produced by stable transfection with the pKS-Hyg plasmid from HS578T cells (HTB 126; LGC Standards GmbH, Wesel, Germany). M13HS-2 and M13HS-8 hybrid cells were derived through spontaneous fusion events between M13SV1-EGFP-Neo cells and HS578T-Hyg cells [23]. All cells were cultured in RPMI 1640 media (PAN Biotech GmbH, Aidenbach, Germany) supplemented with 10% fetal calf serum (PAN Biotech GmbH, Aidenbach, Germany) and 100 U/ml penicillin/ 0.1 mg/ml streptomycin (PAN Biotech GmbH, Aidenbach, Germany) in a humidified atmosphere at 37 °C and 5% CO_2_. The medium was supplemented with the following cell line specific supplements: 10 µg/ml recombinant human epidermal growth factor, 5 µg/ml human recombinant insulin, 0.5 µg/ml hydrocortisone, 4 µg/ml human transferrin, 10 nM β-estrogen, and 400 µg/ml G418 (M13SV1-EGFP-Neo); 200 µg/ml Hygromycin (HS578T-Hyg); 400 µg/ml G418 and 200 µg/ml Hygromycin (M13HS-2 and − 8 hybrid cells). All supplements were purchased from Merck KGaA (Darmstadt, Germany) and PAN Biotech (Aidenbach, Germany).

### XTT proliferation assay

The cells were seeded in triplicates in a 96-well plate and cultured for 24 h in cell line specific medium (5,000 cells/ well). Then, the medium was removed and changed to medium that containing either 0 µg/ml, 5 µg/ml, 10 µg/ml, 25 µg/ml, or 50 µg/ml minocycline (Abcam Limited, Cambridge, UK). Cells were further cultivated for 24 h, 48 h and 72 h in a humidified atmosphere at 37 °C and 5% CO_2_. The medium was subsequently removed, and the wells were filled with 250 µl of RPMI 1640 without phenol red (Pan Biotech, Aidenbach, Germany) and 50 µl of XTT/PMS solution (50 µg/well XTT and 0.25 µg/well PMS; both compounds from Merck KGaA, Darmstadt, Germany). Plates were incubated at 37 °C for three hours, and an ELISA reader (BioTek Instruments, Inc., Winooski, VT, USA) was used to measure the reaction. Three experiments have been conducted independently. Every cell line’s relative proliferation rate was determined in relation to the 24 h value, which was set to 1.

### Western blot analysis

Western blot samples were prepared as described [11]. Briefly, cells (0.5 to 1 × 10^6^) were cultured in a humidified atmosphere at 37 °C and 5% CO_2_. When cells reached a confluency of about 80%, medium was removed and replaced by medium containing 10 µg/ml minocycline. The cells were collected after 24 h, 48 h and 72 h, centrifuged (10 min, 340 × g) and then resuspended in ice-cold RIPA buffer (50 mM Tris-HCl pH 8.0;, 150 mM NaCl, 1% (v/v) NP-40, 0.5% (w/v) sodium deoxycholate, 0.1% (w/v) sodium dodecyl sulphate) supplemented with Pierce Phosphatase Inhibitor Mini Tablets (Thermo Fisher Scientific GmbH, Schwerte, Germany) and cOmplete, Mini, EDTA-free Protease Inhibitor Cocktail (Merck KGaA, Darmstadt, Germany). The samples were lysed by sonification (three times 10 s “on” and 30 s “off”). Subsequently, the total protein concentration was determined using the Pierce™ BCA Protein Assay Kit (ThermoFisher Scientific GmbH, Schwerte, Germany) in accordance with the manufacturer’s instructions. The samples were then mixed with 3 × Laemmli Sample Buffer and denatured by boiling them for 6 minutes at 95 °C. Protein fraction (40 µg/lane) were separated by 10–15% sodium dodecyl sulphate-polyacrylamide gel electrophoresis (SDS-PAGE) and subsequently transferred to an Immobilon polyvinyl difluoride membrane (Merck KGaA, Darmstadt, Germany) under semi-dry conditions. Membranes were incubated for 1 h at room temperature in 5% (w/v) BSA or 5% (w/v) non-fat milk powder (Applichem, Darmstadt, Germany) in Tris-buffered saline (TBS) with 0.1% (v/v) Tween 20 (PBS-T). The following antibodies were used in this study: E-Cadherin (clone 24E10; #3195S), Snail (clone C15D3; 3879 S), Vimentin (#3932S), Sox9 (clone D8G8H; #82630), Slug (clone C19G7; #9585), β-actin (clone AC-15; A5441), anti-mouse IgG, HRP linked (7076 S) and anti-rabbit IgG, HRP linked (7074 S). The β-actin antibody was purchased from Merck KGaA, Darmstadt, Germany. All other antibodies were bought from Cell Signaling Technology Europe B.V., Frankfurt am Main, Germany. Primary antibodies were incubated overnight at 4 °C and secondary antibodies were incubated for 1 h at room temperature (RT). The antibodies were used in the appropriate dilution according to the manufacturer’s specifications. Bands were visualized by using the Aequoria Macroscopic Imaging System (Hamamatsu Photonics Germany, Herrsching am Ammersee, Germany) and the Pierce ECL Western Blot substrate (Thermo Fisher Scientific, Wesel, Germany) as referred to the manufacturer’s manual.

### Zymography

Zymography was performed as described [10]. In brief, cells were plated at a density of 5 × 10^5^ cells/well in a 6-well plate and cultivated for 24 h with 0 µg/ml, 5 µg/ml, 10 µg/ml, and 25 µg/ml minocycline. The supernatants were subsequently collected and mixed with non-reducing Laemmli sample buffer (250 mM Tris-HCL (pH 6.8), 10% (w/v) SDS, 25% (v/v) glycerol, 0,01% (w/v) bromophenol blue) without boiling. Samples were separated using a 10% SDS-PAGE, which was supplemented with 0.1% gelatin (Merck KGaA, Darmstadt, Germany). Thereafter, the gel was washed four-times with washing buffer (50 mM Tris-HCl (pH 7.5), 10 mM CaCl_2_, 2.5% (v/v) Triton X-100, 0.02% NaN_3_) at room temperature for 2 h to remove SDS. Subsequently, the gel was incubated overnight at 37 °C in incubation buffer (50 mM Tris-HCl (pH 7.5), 150 mM NaCl, 10 mM CaCl_2_, 0.02% NaN_3_). Afterwards, the gel was first stained for one hour at room temperature with Coomassie brilliant blue R-250 (Merck KGaA, Darmstadt, Germany) with gentle agitation. Finally, the gel was destained with a destaining solution (45% (v/v), 10% (v/v) acetic acid in H_2_O) until clear bands appeared. Densitometric analysis (Fiji; Image J; https://Fiji.sc) was used to quantify the bands in relation to β-actin expression, which was determined through Western Blot analysis.

### Colony formation assay

The colony formation assay was carried out as described [25]. Cells (500/well) were seeded in duplicates for each condition (0 µg/ml, 5 µg/ml, 10 µg/ml, and 25 µg/ml minocycline). The cells were thencultured for 24 h in a humidified atmosphere at 37 °C and 5% CO_2_. Subsequently, the medium was replaced with medium containing either 0 µg/ml, 5 µg/ml, 10 µg/ml, or 25 µg/ml minocycline. The cells were then cultured for ten more days in a humidified atmosphere at 37 °C and 5% CO_2_. The medium was then removed and the cells were washed twice with PBS. Subsequently, the cells were fixed with 4% paraformaldehyde solution (Merck KGaA, Darmstadt, Germany) for 15 min at room temperature. The fixed cells were washed twice with PBS and subsequently stained with 0.5% crystal violet solution (Merck KGaA, Darmstadt, Germany) for 30 min at room temperature. Finally, plates were thoroughly rinsed with water and dried in the air. The Cell Counter PlugIn for Fiji (Image J; https://Fiji.sc) was used to manually count the formed colonies after plates were scanned.

### Scratch/wound-healing assay

The scratch/wound-healing assay was carried out as described [25]. In short, cells were seeded in triplicates (2 × 10^5^ cells/ well) in a 24-well plate (Sarstedt AG & Co KG, Nümbrecht, Germany) and cultured in a humidified atmosphere at 37 °C and 5% CO_2_ until they reached 100% confluency. To establish the scratch/wound, a 100 µl pipette tip was used. To get rid of dead cells and cell debris, the cells were washed once with PBS. Then, 1.5 ml of fresh media containing 0 µg/ml, 5µg/ml, 10 µg/ml, and 25 µg/ml, respectively, minocycline was added. The 24-well plate was then placed in the Incucyte^®^ SX5 LiveCell Imaging system (Sartorius Lab Instruments GmbH, Göttingen, Germany). Transmission light images were captured every four hours for a total of 24 h at 37 °C and 5% CO_2_. The closure of the scratch/wound was determined using the Fiji software (Image J; https://Fiji.sc). The scratch/ wound areas at t = 4 to 20 h were determined in relation to the scratch/ wound area at t = 0 h, which was set to 100%.

### Statistical analysis

For statistical analysis, the GraphPad PRISM software 8.4.2 (graphpad.com) was used. Data are presented as mean ± standard error of the mean (S.E.M.). p-values were determined using a two-way analysis of variance (ANOVA) and a Tukey’s post-hoc test. Significant differences between the groups were found at the following p-values: * = *p* < 0.0332, ** = *p* < 0.0021, *** = *p* < 0.0002, **** = *p* < 0.0001.

## Results

### Minocycline significantly reduced cell proliferation in a dose dependent manner

**Fig. 1:**
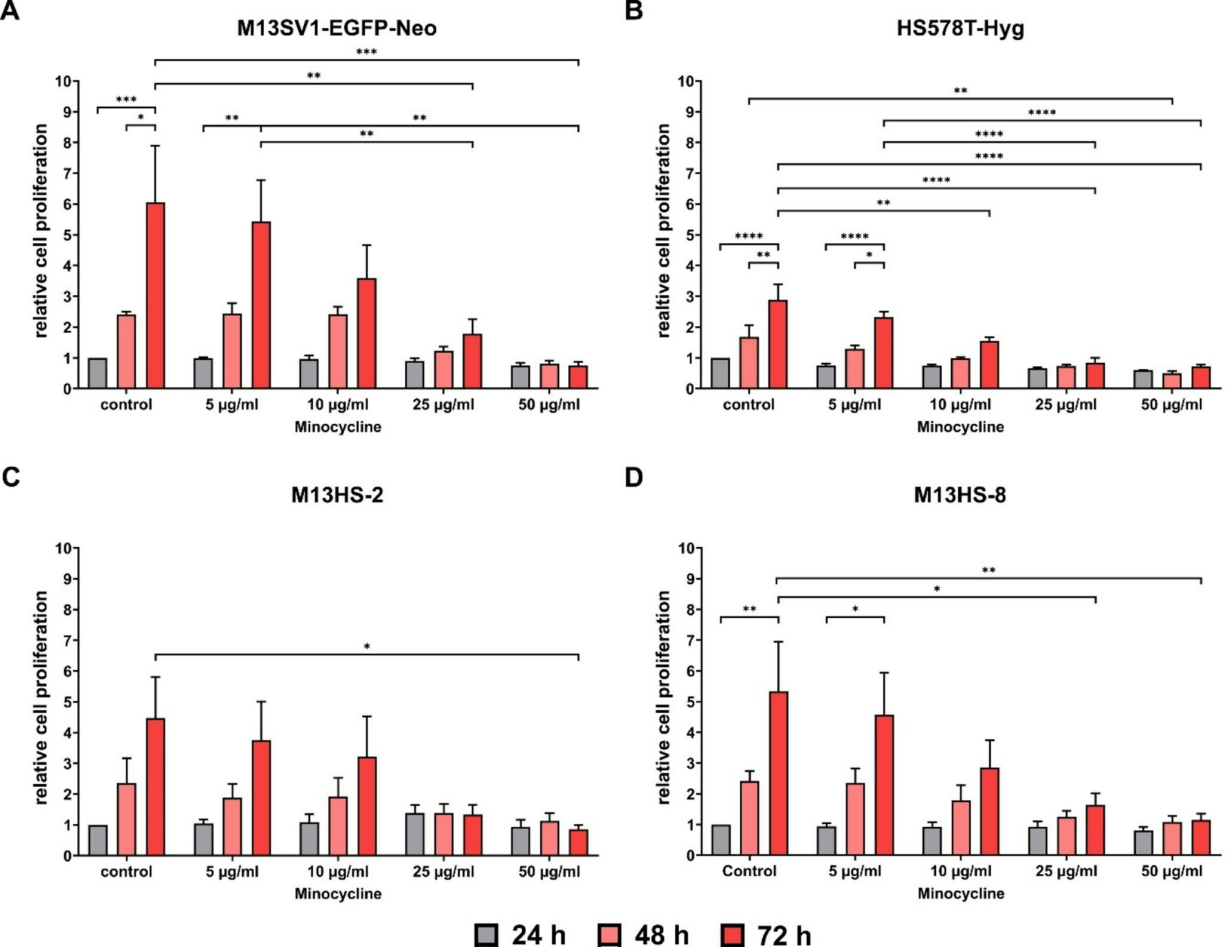
Minocycline significantly inhibits cell proliferation in a dose dependent manner. Cell proliferation was analyzed by XTT assay. The relative proliferation of the cells was calculated in relation to 24 h, which was set to 1. **A)** M13SV1-EGFP-Neo, **B)** HS578T-Hyg, **C)** M13HS-2, **D)** M13HS-8. Shown are the mean ± S.E.M. of at least three independent measurements. Statistical significance was calculated by applying a two-way ANOVA with Tukey post-hoc test. * = *p* < 0.0332, ** = *p* < 0.0021, *** = *p* < 0.0002 and **** = *p* < 0.0001

First, the proliferation of M13SV1-EGFP-Neo, HS578T-Hyg, M13HS-2 and M13HS−8 cells was examined in relation to different concentrations of minocycline (0 µg/ml, 5 µg/ml, 10 µg/ml, 25 µg/ml, and 50 µg/ml). As shown Fig. 1, minocycline inhibited the proliferation of all cell lines in a dose-dependent manner. Moreover, minocycline exhibited a comparable susceptibility to all cell lines. A weak, but still detectable cell proliferation was observed for all cell lines in the presence of 10 µg/ml minocycline (Fig. 1a-d). In contrast, the proliferation of all cell lines was markedly reduced or even absent in the presence of 25 µg/ml and 50 µg/ml minocycline (Fig. 1a-d), which is consistent with earlier research [10]. Interestingly, the proliferation of M13HS-2 hybrid cells was only moderately impaired by 5 µg/ml and 10 µg/ml minocycline in comparison to the other cell lines (Fig. 1c). It is unclear whether this observation was related to the suggested increased drug resistance of tumor hybrids [13–16] which remains to be clarified. However, there was no evidence of an increased minocycline resistance in M13HS-8 tumor hybrids.

### Minocycline impaired the capacity to establish colonies in a dose-dependent manner

**Fig. 2:**
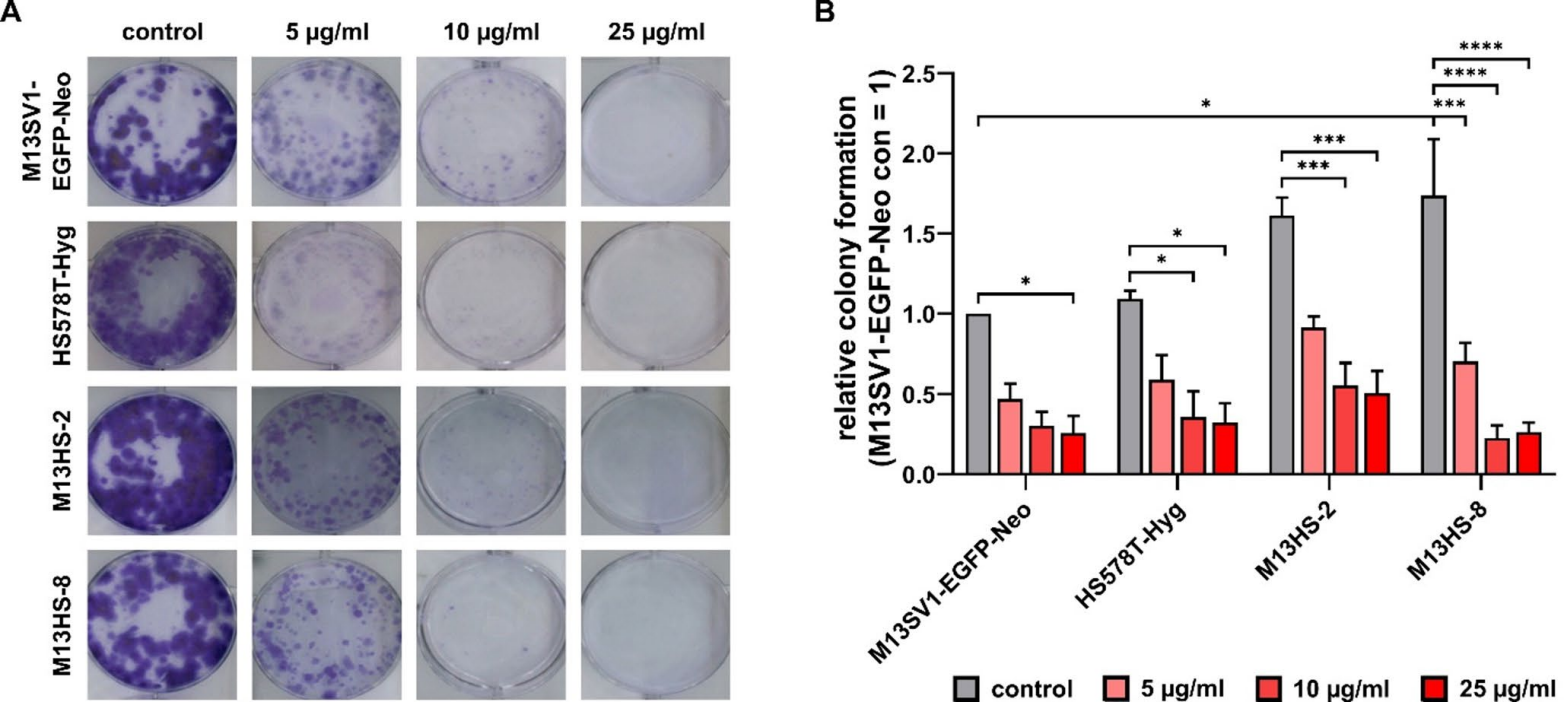
Minocycline inhbits the formation of colonies in a dose-dependent manner. The colony formation assay was used to study the impact of minocycline on the cells ability to form colonies. **(A)** Representative images of colony formation assay results, **(B)** relative colony formation capacity in relation to M13SV1-EGFP-Neo cells, which were set to 1. Shown are the mean ± S.E.M. of at least three independent measurements. Statistical significance was calculated by applying a two-way ANOVA with Tukey post-hoc test. * = *p* < 0.0332, *** = *p* < 0.0002 and **** = *p* < 0.0001

We then looked into whether minocycline affected the ability of M13SV1-EGFP-Neo, HS578T-Hyg, M13HS-2 and M13HS-8 cells to form colonies. The proliferation data showed that 50 µg/ml minocycline stopped cell growth, so this concentration was not used in any more experiments. In accordance with previous data [25], M13HS-2 and − 8 hybrid cells exhibited the highest colony formation capacity (Fig. 2a, b). Similar to the proliferation data, we saw that minocycline decreased the ability of all cell lines to form colonies in a dose-dependent manner (Fig. 2a, b). Interestingly, and contrary to the cell proliferation data, 5 µg/ml minocycline already effectively reduced the colony formation capacity of all cell lines(Fig. 2b). As shown in Fig. 1, the proliferation of all cell lines was only moderately impaired by 5 µg/ml minocycline. Similarly, while data on cell proliferation data show that all cell lines continued to proliferate when exposed 10 µg/ml minocycline (Fig. 1a-d), the capacity of cell lines to form colonies was markedly impaired by 10 µg/ml minocycline and more akin to 25 µg/ml minocycline (Fig. 2b).

### Treatment with Minocycline has different effects on MMP-2 and MMP-9 expression

**Fig. 3:**
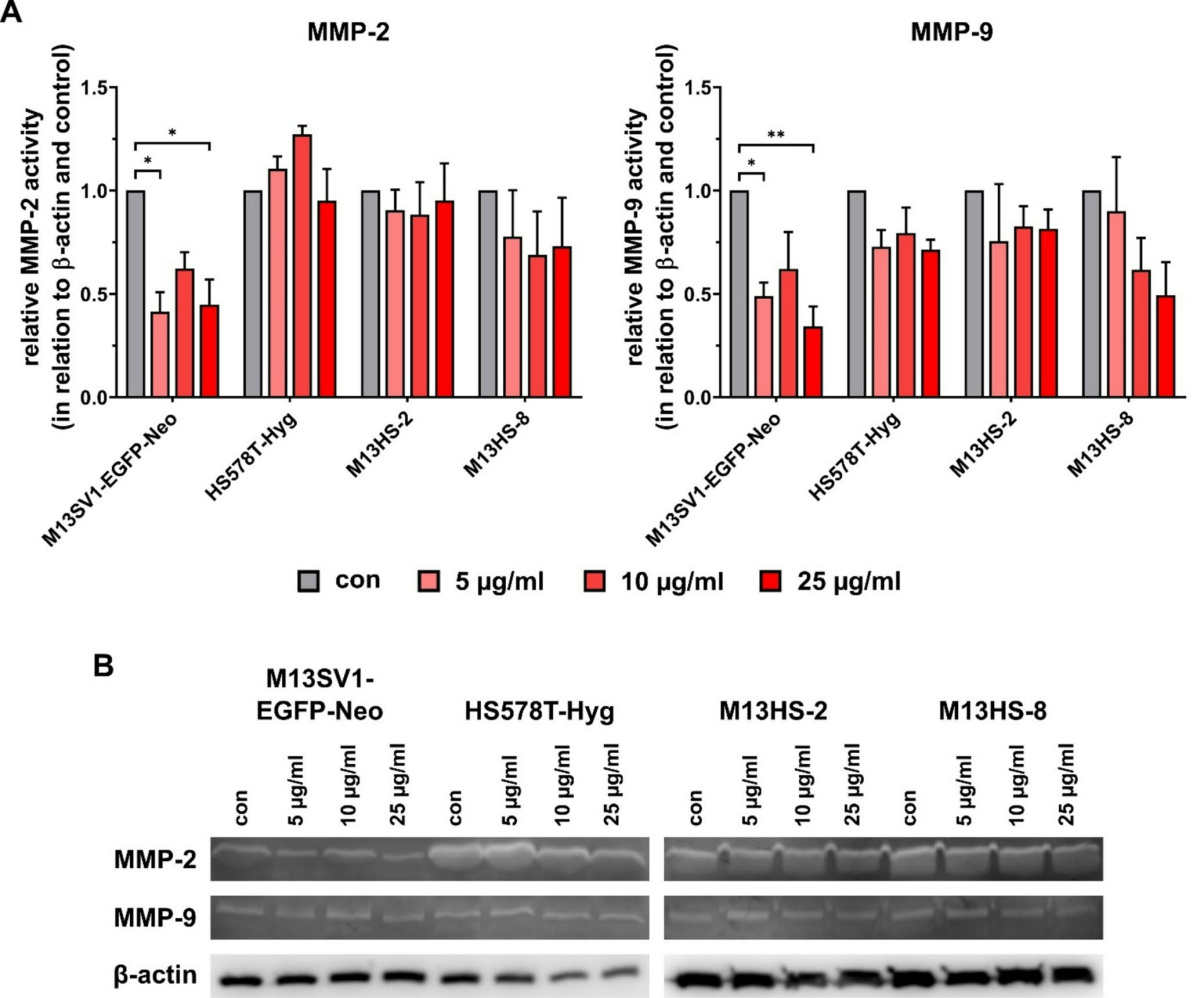
MMP-2 and MMP-9 expression are differently affected by minocycline treatment. The relative expression and activity of MMP-2 and MMP-9 expression in dependence of different minocycline concentrations was measured by zymography and densitometric analysis. **(A)** Relative MMP-2 and MMP-9 activity in relation to β-actin loading control and untreated cells, which were set to 1. Shown are the mean ± S.E.M. of at least three independent measurements. **(B)** Representative MMP-2 and MMP-9 Zymography data. Statistical significance was calculated by applying a two-way ANOVA with Tukey post-hoc test. * = *p* < 0.0332 and ** = *p* < 0.0021

After demonstrating that minocycline treatment impaired the TNF-α induced MMP-9 expression in M13SV1-Cre cells [10, 11], we used zymography to analyze the MMP-2 and MMP-9 expression and activity pattern in M13SV1-EGFP-Neo, HS578T-Hyg, M13HS-2 and M13HS-8 cells. Interestingly, cell line specific variations were observed (Fig. 3). For instance, the expression and activity of MMP-2 and MMP-9 were significantly reduced in M13SV1-EGFP-Neo cells treated with minocycline, but the decrease was not dose-dependent (Fig. 3a). In contrast, MMP-2 expression and activity levels stayed about the same in HS578T-Hyg and M13HS-2 cells. On the other hand, MMP-9 expression and activity levels were marginally, but not significantly reduced in both cell lines (Fig. 3a). In M13HS-8 cells, MMP-9 expression and activity levels were decreased in a dose-dependent manner (Fig. 3a); however the reduction was not significant. MMP-2 expression and activity levels were also diminished in M13HS-8 cells, but this effect was not very strong and there was also no effect that changed with dose (Fig. 3a).

### Minocycline had no effect on the stemness and EMT markers’ expression patterns

**Fig. 4:**
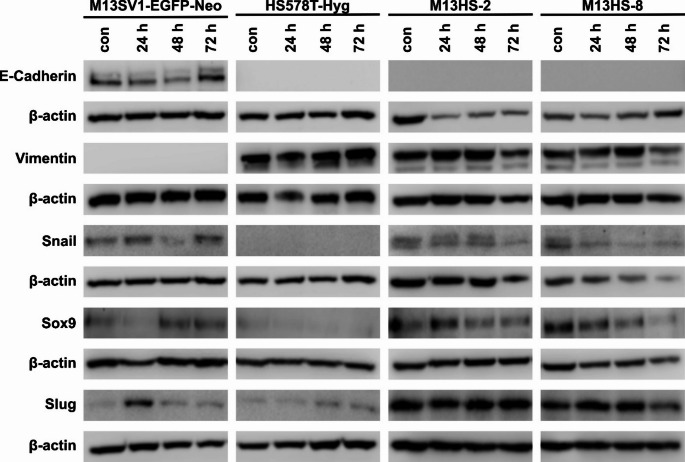
Minocycline has no effect on the expression pattern of EMT and stemness markers. E-Cadherin, Snail and Vimentin were used as EMT markers, whereas Sox9 and Slug were chosen as stemness markers. Shown are representative Western Blot data of at least three independent experiments. Please note that the β-actin loading control for E-Cadherin and Snail expression of M13SV1-EGFP-Neo cells and HS578T-Hyg cells are identical since both target proteins were probed on one Western blot

More research was done to see if minocycline changed the expression patterns of EMT and stemness markers in the cells over time. Thus, cells were administered to 10 µg/ml minocycline at three different time points (24 h, 48 h and 72 h). The reason for this is that the cells’ proliferation was markedly but not entirely suppressed by 10 µg/ml minocycline (Fig. 1), allowing them to be cultivated for up to 72 h. M13SV1-EGFP-Neo breast epithelial cells showed a classical epithelial expression pattern: they expressed E-Cadherin, but not Vimentin (Fig. 4), which is in line with previous results [25]. Additionally, Snail, Sox9 and Slug were expressed by the cells (Fig. 4). In contrast, HS578T-Hyg breast cancer exhibited a classical mesenchymal phenotype as evidenced by the absence of E-Cadherin and Snail expression and the presence of Vimentin (Fig. 4), which is also consistent with prior research [25]. The M13HS-2 and M13HS-8 hybrid cells also exhibited a classical mesenchymal phenotype, as they were Vimentin positive but E-Cadherin negative (Fig. 4). The expression levels of Vimentin in M13HS-2 and M13HS-8 tumor hybrids were comparable to those of HS578T-Hyg cells. In line with previous work [25], both hybrid cells also expressed Snail. Western blot data suggest that the expression of Sox9 and Snail M13HS-2 and M13HS-8 tumor hybrids treated with minocycline was likely reduced (Fig. 4). However, the densitometric analysis of all Western blot data did not reveal any differences in the overall expression pattern of the selected EMT and stemness markers between non-treated and minocycline treated cells (Supplemental data 1).

### Minocycline inhibited the migration of cells

**Fig. 5:**
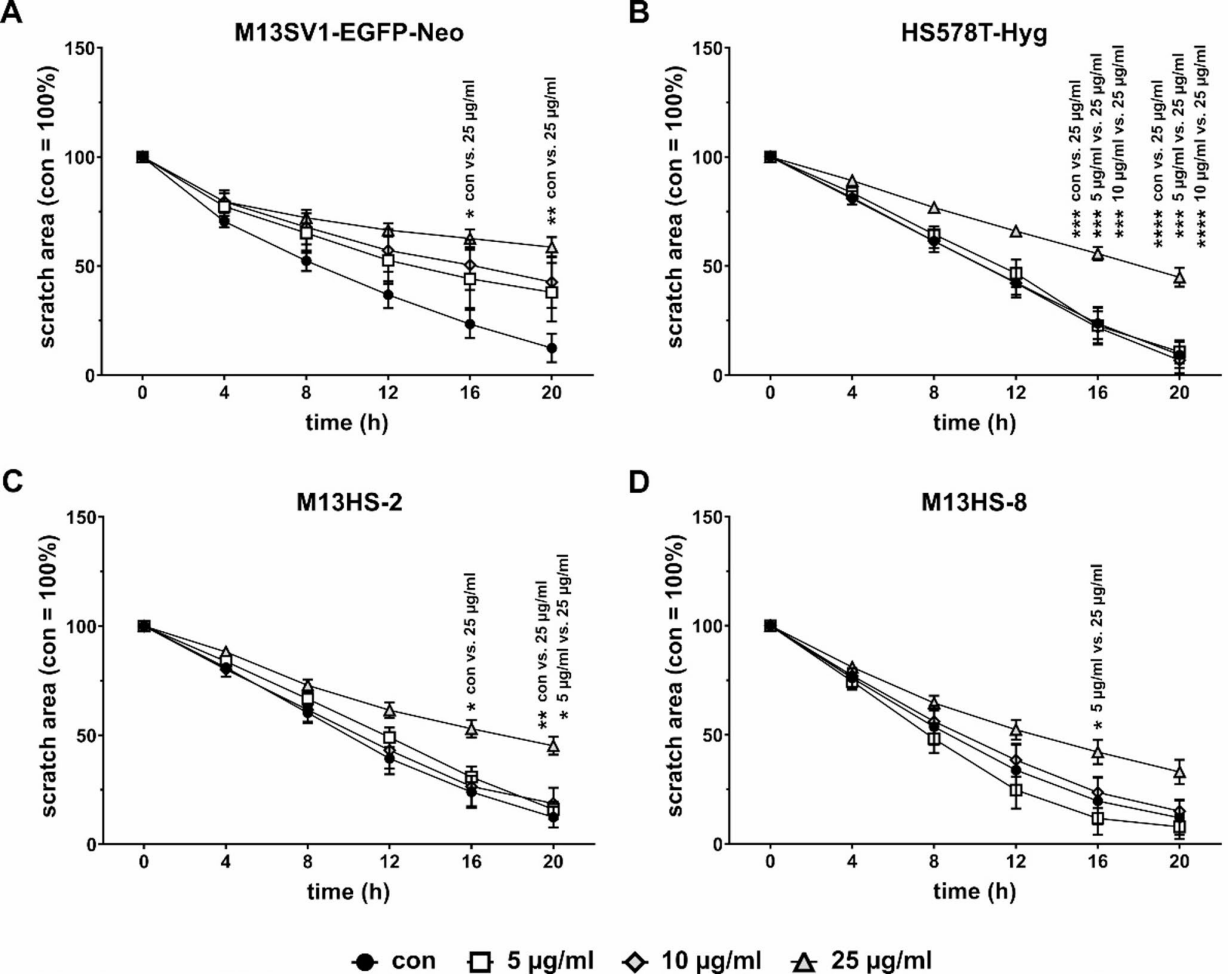
Minocycline impairs cell migration. Cell migration was analyzed by using the Scratch/wound healing assay and the Incucyte^®^ SX5 LiveCell Imaging system. The closure of the scratch area was determined in relation to the scratch size at t = 0 h, which was set to 100%. **(A)** M13SV1-EGFP-Neo, **(B)** HS578T-Hyg, **(C)** M13HS-2, **(D)** M13HS-8. Shown are the mean ± S.E.M. of at least three independent experiments. Statistical significance was calculated by applying a two-way ANOVA with Tukey post-hoc test. * = *p* < 0.0332, ** = *p* < 0.0021, *** = *p* < 0.0002 and **** = *p* < 0.0001

Next, a Scratch/wound healing assay was used to evaluate the migration of cells in response to 0 µg/ml, 5 µg/ml, 10 µg/ml, and 25 µg/ml minocycline. The results are shown in Fig. 5, which clearly show that minocycline inhibited cell migration. Interestingly, minocycline inhibited the migration of M13SV1-EGFP-Neo cells in a dose-dependent manner (Fig. 5a). Conversely, only the highest concentration of minocycline, 25 µg/ml, (significantly) inhibited the migration of HS578T-Hyg breast cancer cells and M13HS-2 and M13HS-8 tumor hybrids (Fig. 5b-d). Notably, minocycline did not have any cytotoxic effects, suggesting that it likely interferes with the cells’ molecular migration machinery. Representative images from the Scratch/wound healing assay can be found in Supplemental data 2.

## Discussion

The reason for this study came from the fact that we already found that minocycline effectively impaired the TNF-α-induced fusion of M13SV1-Cre human breast epithelial cells and MDA-MB-435-pFDR1 human cancer cells [10, 11]. Furthermore, minocycline has been suggested as a potential drug in cancer treatment [1, 2]. Consistent with published data [10, 26], the obtained data demonstrated that minocycline inhibited the tested cell lines’ ability to proliferate as well as to form colonies in a dose-dependent manner. Interestingly, Pourgholami et al. observed that 10 µmol/ml minocycline (equal to 4.57 µg/ml) had only a moderate effect on the ability of OVCAR3, SKOV-3 and A2780 ovarian carcinoma cells to form colonies [26], which is different from our results. As shown in this study, a concentration of 5 µg/ml (equal to 10.93 µmol/l) minocycline was found to be sufficient to inhibit the colony formation capacity of all tested cell lines (Fig. 2). However, higher concentrations of minocycline (50 µmol/l (ca. 22.9 µg/ml) and 100 µmol/l (ca. 45.7 µg/ml)) completely abrogated the ability of OVCAR3, SKOV-3 and A2780 ovarian carcinoma cells to form colonies [26], which is consistent with our data. Liu and colleagues’ findings likewise demonstrated that 50 µmol/l minocycline inhibited the colony formation capacity of C6 glioma cells by 60 [27]. Conversely Pourgholami et al. findings indicated that 50 µmol/l minocycline completely abrogated the colony formation capacity of ovarian carcinoma cells [26]. We assume that cell line specific differences were the most likely cause of these discrepancies. The cell proliferation and colony formation data indicated that all cell lines exhibited differential susceptibilities to 5 µg/ml minocycline. Although the proliferation of cells was only moderately affected, the colony formation capacity was markedly diminished by 5 µg/ml minocycline. At present, we are unable to provide a satisfactory explanation for this finding. We hypothesize that these discrepancies were most likely caused by differing minocycline to cell number ratios (proliferation: 5 µg/ml minocycline to 15,625 cells/ cm^2^ vs. colony formation: 5 µg/ml minocycline to 52 cells/ cm^2^) and to different incubation times (cell proliferation: up to 72 h vs. colony formation: 14 days).

Surprisingly, Western blot data revealed that 72 h treatment with 10 µg/ml minocycline did not change the expression of EMT markers in all cell lines. Increasing evidence has suggested that NF-κB signaling is involved in the induction and maintenance of invasive phenotypes that are associated with EMT and metastasis [28, 29]. Minocycline is a well-known NF-κB inhibitor [11, 30]. As a consequence, the prolonged treatment of cells with 10 µg/ml minocycline should have resulted in decreased expression levels of EMT markers. In fact, Yang and colleagues demonstrated that minocycline inhibited EMT in SW480 and SW620 human colon cancer cell lines [8]. Interestingly, the expression of N-Cadherin, Vimentin, Slug, and Snail was down-regulated in both colon cancer cell lines at concentrations of 4 µM and 8 µM of minocycline [8]. Unfortunately, the authors did not specify the duration of minocycline treatment for the cells [8]. In any case, the concentrations of 4 µM and 8 µM minocycline are equal to 1.8 µg/ml and 3.6 µg/ml, respectively, which are lower than the concentrations that were used in this study. In contrast, we have previously demonstrated that the TNF-α induced MMP-9 expression in M13SV1-Cre cells was sufficiently affected by 10 µg/ml minocycline due to inhibition of NF-κB activity [10]. Since both M13SV1-EGFP-Neo and M13SV1-Cre cells were derived from the same M13SV1 parental cell line, we think that both stable transfected M13SV1 variants are equally susceptible to minocycline. Thus, the minocycline-dependent reduction in MMP-2 and MMP-9 expression levels in M13SV1-EGFP-Neo cells may also be attributed to inhibition of NF-κB activity. Nevertheless this has to be confirmed in future studies.

It is still unclear as to why the expression levels of MMP-2 and MMP-9 in HS578T-Hyg breast cancer cells and M13HS-2 and − 8 tumor hybrids remained unaltered or only moderately decreased. Rezaei and colleagues demonstrated that the expression levels of MMP-2 and MMP-9 were suppressed in minocycline-treated MCF-7 breast cancer cells [12]. It might be speculated that the 10 µg/ml concentration of minocycline wasn’t high enough to impair protein expression in these cell lines, even though the proliferation of cells was sufficiently blocked by 10 µg/ml minocycline. This assumption is consistent with the findings of Rezaei et al., who showed that the expression of MMP-2 and MMP-9 was significantly down-regulated in MCF-7 breast cancer cells when exposed to 36.10 µM minocycline [12]. Furthermore, these findings may also suggest that different cell lines or tissues are more or less sensitive to minocycline. The results of the Scratch/wound healing assay support this assumption. The migration of M13SV1-EGFP-Neo breast epithelial cells was inhibited in a dose-dependent manner by minocycline. Conversely, the migration of HS578T-Hyg breast cancer cells and M13HS-2 and M13HS-8 tumor hybrids was impaired by 25 µg/ml minocycline. Interestingly, the results of Scratch/wound healing assay also demonstrated that 5 µg/ml minocycline inhibited the migration of M13HS-2 tumor hybrids more effectively than the migration of M13HS-8 cells. The migratory behavior of untreated M13HS-2 and M13HS-8 tumor hybrids is comparable, which is in accordance with recent data [25].

It is possible that cell line-specific differences are the cause of the observed differences between the M13HS-2 and M13HS-8 tumor hybrids in the presence of 5 µg/ml minocycline. The finding that minocycline impaired the migration of M13SV1-EGFP-Neo breast epithelial cells, HS578T-Hyg breast cancer cells and M13HS-2 and M13HS-8 tumor hybrids is consistent with the results of Yang and colleagues [8] and Brundula and colleagues [3]. In these studies, minocycline significantly inhibited the migration of leukocytes, and SW480 and SW620 colon cancer cells [3, 8]. While Brundula et al. came to the conclusion that minocycline blocked the transmigration of leukocytesdue to markedly decreased MMP-2 and MMP-9 expression levels [3], Yang et al. assumed that minocycline inhibited the migration and invasion of colon cancer cells by directly binding the Src kinase Lyn [8]. Notably, the zymography data revealed that the MMP-2 and MMP-9 expression levels were lower in M13SV1-EGFP-Neo cells that were treated with minocycline. This finding is consistent with the reduced migration activity of the cells in the presence of minocycline and the data of Brundula and colleagues [3]. This is further supported by the study of Rezaei and colleagues, which demonstrated that minocycline significantly reduced the expression of MMP-2 and MMP-9 as well as the migratory activity of MCF-7 breast cancer cells [12]. Various studies have demonstrated that minocycline inhibits the growth of cancer cells by inducing apoptosis [12, 26, 27]. Hence, it might be speculated that the observed reduced cell migration in this study was more likely due to cell death. However, the Scratch/wound healing assay images did not indicate an increased induction of apoptosis in minocycline treated cells. Therefore, we conclude that minocycline directly interfered with cell migration specific signal transduction cascades, thereby inhibiting the migration of M13SV1-EGFP-Neo, HS578T-Hyg, M13HS-2 and M13HS-8 cells.

## Conclusion

In conclusion, our results show that the antibiotic minocycline elicited distinct responses in M13SV1-EGFP-Neo breast epithelial cells, HS578T-Hyg breast cancer cells and M13HS-2 and M13HS-8 tumor hybrids. Minocycline inhibited the proliferation of all cell lines and their capacity to form colonies in a dose-dependent manner by minocycline, but it had no effect on the cells’ EMT and stemness expression pattern. Similarly, the cell migration and zymography data demonstrated that the cell lines were susceptible to varying concentration of minocycline. For instance, M13SV1-EGFP-Neo breast epithelial cells were more susceptible to lower minocycline concentrations than HS57T-Hyg cells and M13HS-2 and M13HS-8 tumor hybrids. Minocycline has been proposed as a potential compound for the treatment if cancer and cancer-related diseases, including breast cancer [1, 2]. On the one hand, 25 µg/ml minocycline significantly inhibited the proliferation, colony formation capacity and migration of HS578T-Hyg breast cancer cells and M13HS-2 and M13HS-8 tumor hybrids. These findings likely support the potential anti-tumor effects of minocycline. However, our data also revealed that minocycline had a comparable effect on non-transformed M13SV1-EGFP-Neo breast epithelial, which may indicate possible unwanted side-effects. Thus, more research is necessary to investigate whether minocycline can be used as a potential medication to treat neoplastic tumor diseases, such as breast cancer and their tumor hybrids.

## Electronic supplementary material

Below is the link to the electronic supplementary material.

Supplementary Material 1

Supplementary Material 2

## Data Availability

No datasets were generated or analysed during the current study.

## Abbreviations

ANOVA: Analysis of variance
EGFP: Enhanced green fluorescent protein
EMT: Epithelial-to-mesenchymal transition
IL-1β: Interleukin-1β
MMP: Matrix metalloproteinases
NF-κB: Nuclear factor-kappa-light-chain-enhancer of activated B cells
RT: Room temperature
SDS-PAGE: Sodium dodecyl sulphate-polyacrylamide gel electrophoresis
S.E.M.: Standard error of the mean
STAT3: Signal transducer and activator of transcription 3
TNF-α: Tumor necrosis factor-α
