# Supplementary figures and images for: Differential effects of minocycline on human breast epithelial cells, human breast cancer cells and their tumor hybrids
Source: Mol Biol Rep. 2025 Jun 5;52(1):553. doi: 10.1007/s11033-025-10666-1 (PMC12141121; doi:10.1007/s11033-025-10666-1)

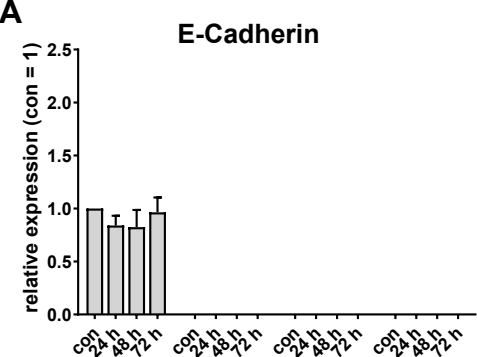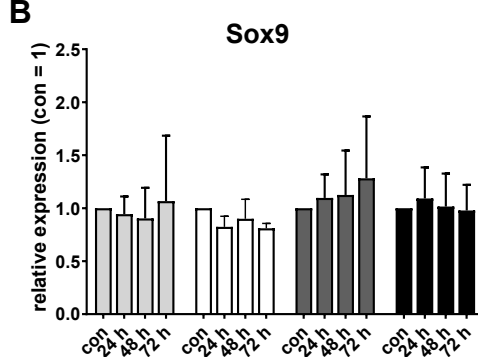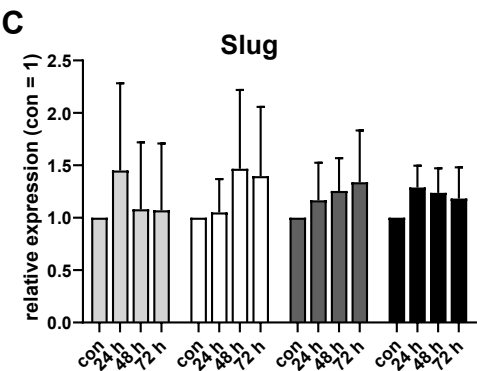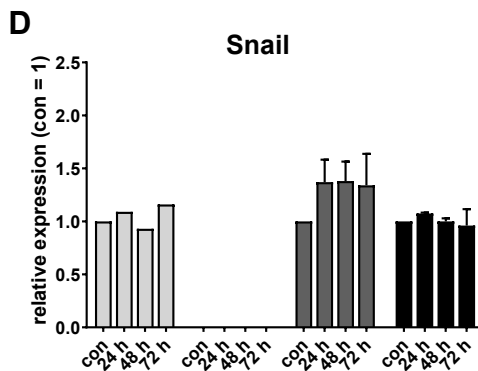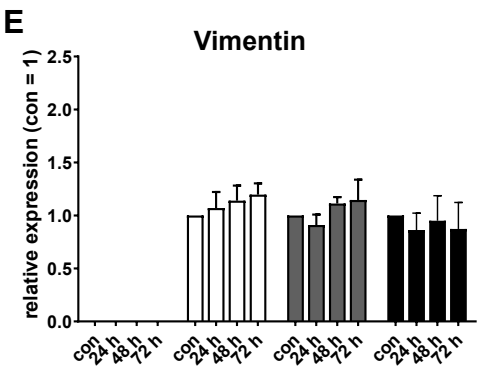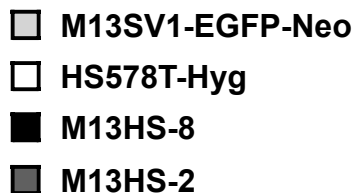

Supplement: Supplementary file 1 — Supplementary Material 1 [file 11033_2025_10666_MOESM1_ESM.pdf]

**M13SV1-EGFP-Neo**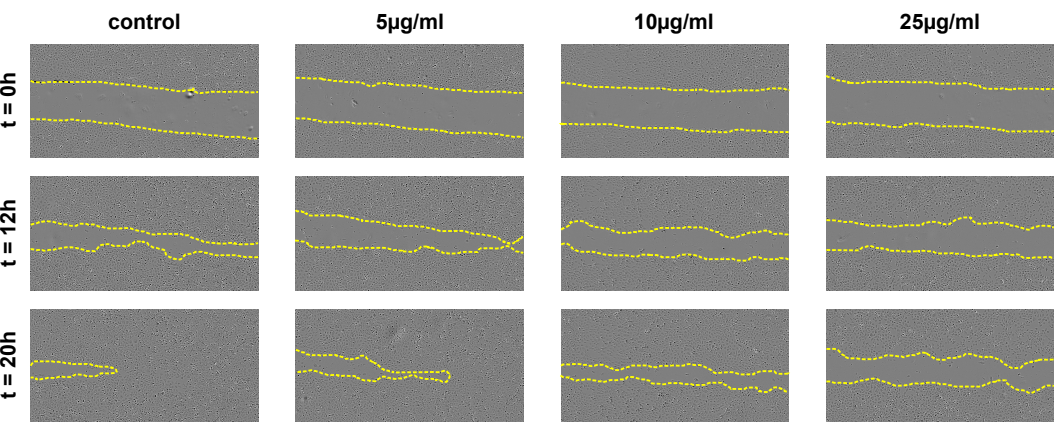**HS578T-Hyg**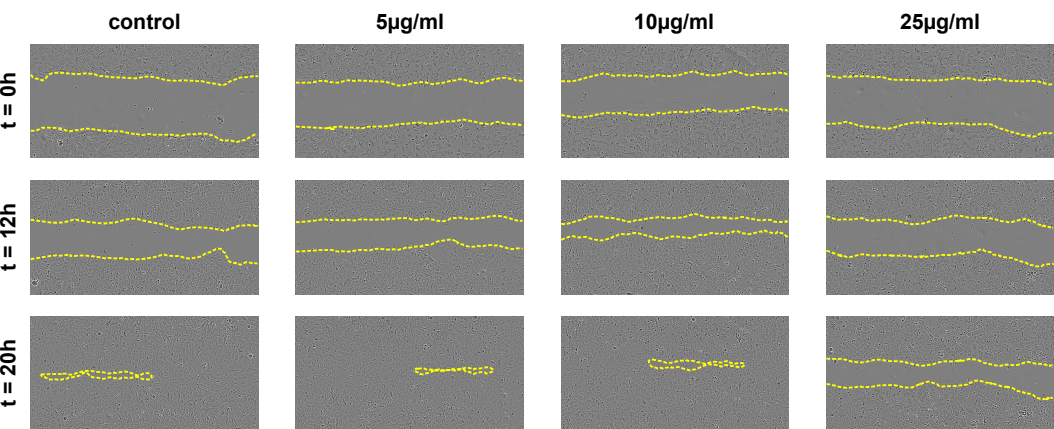**M13HS-2**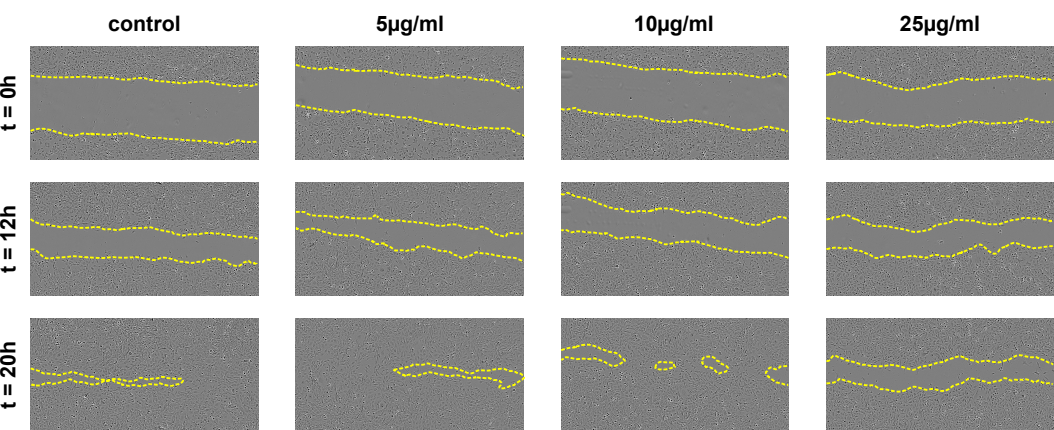**M13HS-8**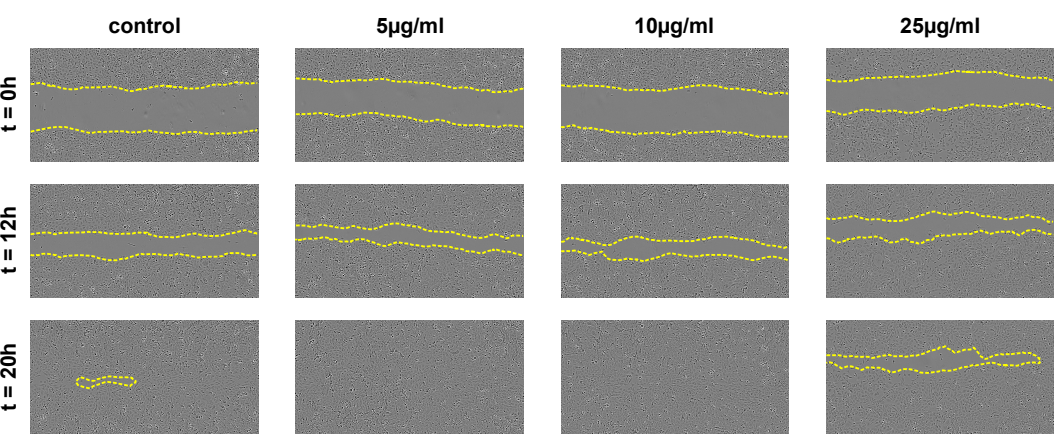

Supplement: Supplementary file 2 — Supplementary Material 2 [file 11033_2025_10666_MOESM2_ESM.pdf]
